# Natural Killer Cells in Anti-Neutrophil Cytoplasmic Antibody-Associated Vasculitis - A Review of the Literature

**DOI:** 10.3389/fimmu.2021.796640

**Published:** 2022-01-14

**Authors:** Sina Fuchs, Andrea Scheffschick, Iva Gunnarsson, Hanna Brauner

**Affiliations:** ^1^ Division of Rheumatology, Department of Medicine, Solna and Center for Molecular Medicine, Karolinska Institutet, Stockholm, Sweden; ^2^ Rheumatology, Karolinska University Hospital, Stockholm, Sweden; ^3^ Dermato-Venereology, Karolinska University Hospital, Stockholm, Sweden

**Keywords:** natural killer cells, anti-neutrophil cytoplasmic antibody, ANCA - associated vasculitis, microscopic polyangiitis (MPA), granulomatosis with polyangiitis (GPA), eosinophilic granulomatosis with polyangiitis (EGPA)

## Abstract

Anti-neutrophil cytoplasmic antibody (ANCA)- associated vasculitis (AAV) is a group of systemic autoimmune diseases characterized by inflammation of small- and medium-sized vessels. The three main types of AAV are granulomatosis with polyangiitis (GPA), microscopic polyangiitis (MPA) and eosinophilic granulomatosis with polyangiitis (EGPA). A growing number of studies focus on natural killer (NK) cells in AAV. NK cells are innate lymphoid cells with important roles in anti-viral and anti-tumor defense, but their roles in the pathogenesis of autoimmunity is less well established. In this review, we will present a summary of what is known about the number, phenotype and function of NK cells in patients with AAV. We review the literature on NK cells in the circulation of AAV patients, studies on tissue resident NK cells and how the treatment affects NK cells.

## Highlights

- The phenotype of circulating NK cells is altered in AAV patients and indicates activation.
- NK cells are found in the inflamed organs but in low numbers.
- The possible role of NK cells in the pathogenesis of AAV remains to be determined.
- NK are influenced by, and potentially contribute, to the effect of medications used in AAV.

## Introduction

Vasculitis is a group of diseases characterized by inflammation of blood vessels. Anti-neutrophil cytoplasmic antibody (ANCA)-associated vasculitis (AAV) is an autoimmune disease with inflammation affecting small- and medium-sized vessels associated with the presence of ANCA (1, 2). AAV can be further subdivided in three diagnostic entities, granulomatosis with polyangiitis (GPA), microscopic polyangiitis (MPA) and eosinophilic granulomatosis with polyangiitis (EGPA) with the presence of ANCA autoantibodies targeting the myeloperoxidase (MPO) or the neutrophil cytoplasmic antigens proteinase 3 (PR3). AAV are systemic diseases that can affect multiple organs including the upper and/or lower respiratory tracts, kidneys, skin and neural tissue and many patients also have common symptoms with fatigue, fever, and musculoskeletal symptoms. Although the different subgroups of AAV share many clinical characteristics, distinct associations to certain HLA-types being either associated to increased risk of disease or shown to be protective have been found (3–8), suggesting that the diseases may be separate entities.

The role of the immune system in the disease pathogenesis of AAV is also well established. Key events include loss of tolerance of T and B cells, followed by production of ANCA and subsequent activation of neutrophils and the destruction of vessels (9). Other immune cells have also implicated to have a role in the disease pathogenesis, including natural killer (NK) cells (10, 11). In this review, we focus on the current knowledge of NK cells in AAV.

## NK Cell Biology and Function

NK cells are innate lymphoid cells that participate in the early anti-viral and anti-tumor defenses by recognizing and eliminating target cells without the need for prior activation (12). In addition to the killing of cancer and infected cells, NK cells can also eliminate otherwise stressed cells including senescent cells, over activated CD4^+^ T cells and autoreactive CD8^+^ T cells (13–15), and a growing number of studies suggest possible roles for NK cells also in autoimmunity. NK cells lack antigen specific receptors, but instead NK cell activation is accomplished by input through activating and inhibiting receptors and the net input determines if the release of perforin and granzyme B and target cell lysis will occur (16). Cytotoxicity by NK can also be performed *via* death receptor-mediated apoptosis (17). NK cells can furthermore eliminate antibody coated cells *via* engagement of the FcγRIIIA (CD16) receptor, leading to antibody dependent cellular cytotoxicity (ADCC) and cytokine production (11). The activation of NK cells *via* CD16 does not require any co-stimulation and is also an important link between NK cells and the adaptive immunity (18).

In addition to cytotoxic functions, NK cells can secrete cytokines like IL-10, TGFβ, TNFα or IFNγ and thereby exhibit immunomodulatory functions, which can either prevent or promote inflammatory processes (19). NK cell activation can also be achieved by a wide range of pro-inflammatory cytokines like IL‐2, IL‐12, IL‐15, IL‐18 or signals through toll-like receptors (TLR´s) (20). Phenotypically, NK cells are characterized by surface expression of the adhesion molecule CD56 and absence of CD3. The percentage of NK cells in healthy human peripheral blood is highly variable, approximately 2 – 30% of total lymphocytes (21, 22). Variations in the phenotypic markers used to define NK cells in the different studies complicate comparisons. For example, older studies are limited by the identification of NK cells only with one phenotypic marker, often CD16, and in studies performed with clinical routine staining CD16 and CD56 are sometimes indistinguishable (23). The majority of NK cells in peripheral blood are CD56^dim^, while CD56^bright^ NK cells are enriched in secondary lymphoid organs, such as lymph node or tonsil, and other tissue including liver (24–26). Classically, CD56^dim^ NK cells are known to be more cytotoxic and stronger activated by cell-cell contact. CD56^bright^ NK cells are rather cytokine producers and more responsive tosoluble factors, including chemokines and cytokines, but both subsets can exert cytotoxicity and release cytokines upon appropriate stimulus (27, 28). Another subset of NK cells, expressing the maturation marker CD57, is characterized by a cytotoxic phenotype but less sensitive in the response to cytokines (29).

## The Role of NK Cells in Autoimmune Diseases

**Figure 1:**
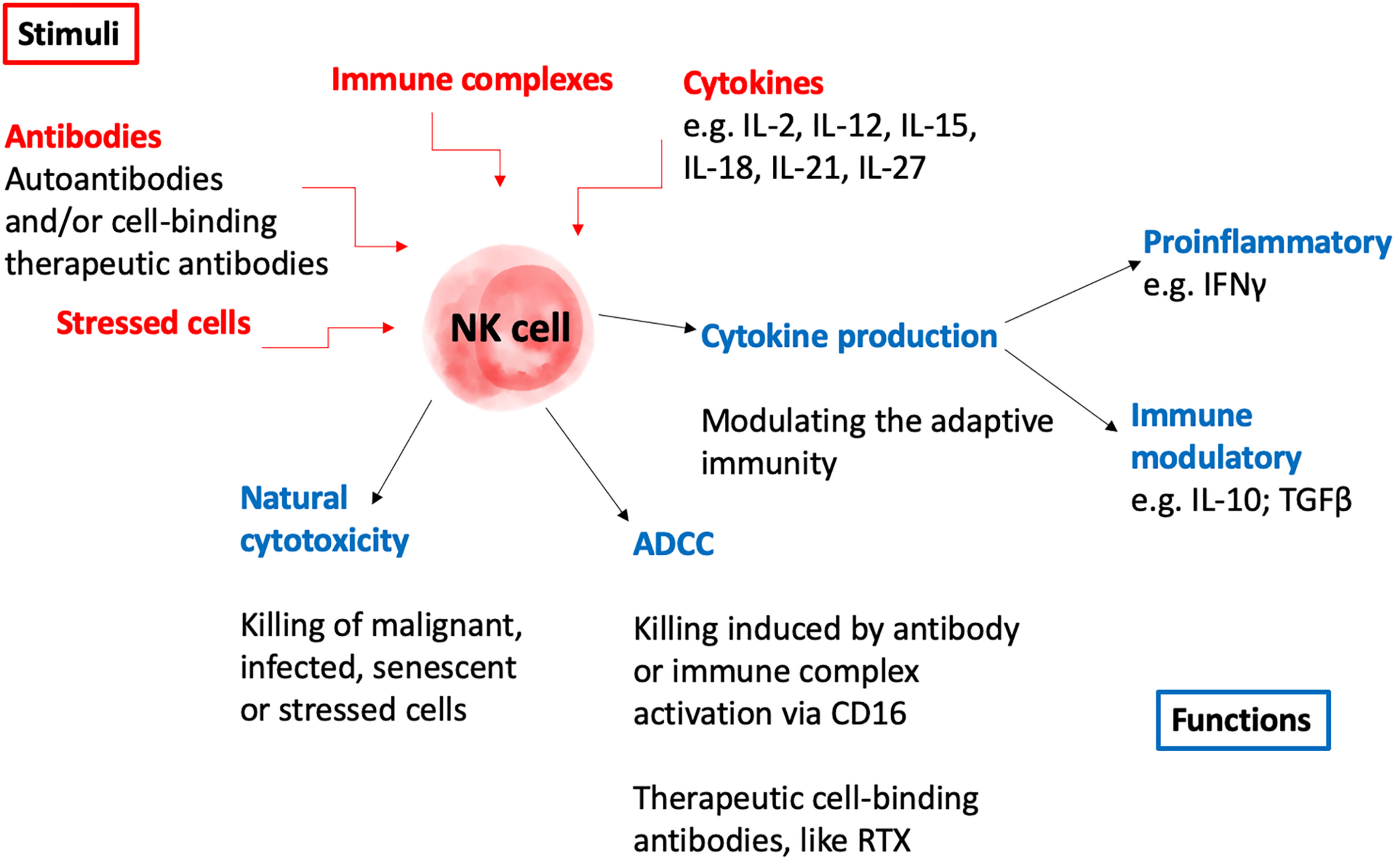
Schematic illustration of potential roles of NK cells in the pathogenesis of autoimmunity. AAV, anti-neutrophil cytoplasmic antibody (ANCA)-associated vasculitis; ADCC, antibody dependent cellular cytoxicity; IFNγ, interferon gamma; IL, interleukin; NK, natural killer; RTX, rituximab; TGFβ, transforming growth factor beta.

The exact role for NK cells in autoimmunity is still controversial and data support both pathogenic and protective functions. On one hand, NK cells could directly participate in destroying host tissue, produce high levels of pro-inflammatory cytokines and kill T regulatory cells that normally prevent autoimmunity (30–32). On the other hand, NK cells do not only have cytotoxic functions, but can also secrete inhibitory cytokines, such as IL-10 and TGFβ, thus leading to a down regulation of the immune system and can block autoreactive CD8^+^ T cells (33). Hence, dysfunctional NK cells can result in an imbalance of the immune response which could both dampen and exacerbate autoimmune disease. NK cells can further participate in cytotoxicity of cells bound to therapeutic antibodies *via* ADCC in patients with autoimmune disease. There are many reviews focusing on the role of NK cells in autoimmunity (34–40), and some examples from different autoimmune diseases are outlined here. NK cell numbers are reduced in the circulation of many autoimmune diseases, including systemic lupus erythematosus (SLE), rheumatoid arthritis (RA), and Sjögren’s syndrome (41, 42). In patients with SLE, little is known about tissue infiltrating NK cells. Recently however, NK cell transcripts were detected by single cell RNA sequencing of kidney tissue from lupus nephritis patients in which they found two NK cell subsets, one with cytotoxic and one with tissue residency properties (43). In a mouse model for type 1 diabetes (T1D) NK cells with a more active phenotype and function were found in the pancreas (44) and in a unique small study of human pancreas sections from recent onset T1D patients half of the analyzed patients displayed cellular infiltrates dominated by NK cells (45). In addition, NK cells are present in actively demyelinating MS lesions in human patients (46) and in patients with RA, NK cells infiltrating inflamed joints were more prone to IFNγ secretion than NK cells from the circulation, indicating a possible disease-promoting function in the target tissue (31). Moreover, NK cells from the synovial fluids have been shown to secrete TNFα and IFNγ, leading to an activation state of synovial fibroblasts expressing HLA-DR^+^CD90^+^, and are considered to be involved in bone destruction (47, 48). Furthermore, a recent study showed an inverse correlation between NK cell numbers in the circulation and disease severity in RA (49). Conclusively, NK cells can exhibit disease-protecting or disease-enhancing properties in autoimmunity and because of the different properties reported on circulating and tissue infiltrating NK cells investigations both in the target organs as well as in the circulation are needed (**Figure 1**).

## NK Cells in AAV

Data on the role of NK cells in AAV are largely lacking. In the present review we included all English written paper identified on PubMed on NK cells in AAV until June 2021, searching for NK cells, innate lymphoid cell 1 and AAV as well as the disease subtypes GPA, MPA and EGPA. We also screened the literature by using the old terms of the diseases, Churg-Strauss syndrome and Wegener’s granulomatosis.

## Peripheral NK Cells in AAV

Several publications describe unaltered NK cell numbers and percentages in peripheral blood mononuclear cells (PBMCs) of AAV patients compared to control (**Table 1**) (52, 54, 58, 59). The literature focusing on NK cell numbers or percentage in AAV is however inconclusive, and some studies instead report a decrease or increase of NK cell numbers or percentage in these patients (23, 53, 55–57), in line with what has been reported for some other autoimmune diseases (60). Also in healthy persons there is however great variations in percentage and absolute numbers of NK cells and high age is generally an important contributing factor that may vary between studies (23). Hypothetically, this incongruence of data might also be explained by differences in the study population but also by the fact that the amount of NK cells in circulation can vary between AAV disease phenotype, different phases of the disease and in relation to treatment. In most studies of peripheral NK cells in AAV the ratio between CD56^bright^ and CD56^dim^ cells was unaltered in AAV compared to controls (52, 54, 55, 59). However, ANCA-negative AAV patients had a lower proportion of CD56^dim^ cells compared to healthy controls whereas the frequency of CD56^bright^ cells and CD56^dim^ cells expressing high levels of CD57 were not influenced by occurrence of ANCA (59). Decreased NK cell numbers, and also specifically CD56^dim^ cells, were further found in the active phase of disease (57) and levels returned to normal in disease remission (56, 57), but these differences were not confirmed in all studies (58). One interesting possibility is that the decrease of NK cells in the circulation in active phase of AAV compared to healthy controls reflects recruitment to the site of inflammation, thus lowering the number in circulation. Since the numbers of circulating NK cells negatively correlate with disease activity in GPA patients (55, 57), one possibility to further explore is if NK cells can act as a marker for disease activity in all AAV patients. The decrease in NK cell numbers during the active phase of disease was also confirmed in an analysis of GPA and MPA patient groups separately, but this study was not able to confirm an increase in remission (56).

**Table 1:** NK cells in circulation of AAV patients.

Reference	Patient group (no. of patients)	NK cell numbers	Phenotype	Function	Therapeutics
**Kottilil et al.** (50)	GPA (19)	n/a	Increased CCR5 expression in active phase compared to remission. Unaltered expression of CXCR4, CD158a, CD158b, CD94, 2B4 and CD161 in active phase and remission compared to control.	n/a	Highly active antiretroviral therapy, containing at least one protease inhibitor and two reverse-transcriptase inhibitors of HIV.
**Miyashita et al.** (51)	MPA (43)	n/a	Decreased frequency of KIR2DS3 compared to control.	n/a	All patients, except 3 patients with MPA and 1 with classic PAN, werereceiving treatment with corticosteroids. Twenty patients with MPA, 6 with CSS, and 2 with WG were being treated with cyclophosphamide, and 1 patient with classic PAN was being treated with methotrexate. This study was reviewed and approved by the ethics committees of the participating institutions 40 out of 43 were receiving treatment with corticosteroids, 20 out of 43 were treated with CYC.
**Tadema et al.** (52)	AAV (38)	Unaltered NK cell numbers and CD56^bright^ and CD56^dim^ NK cells compared to control.	Increased expression of TLR 2, 4 and 9 compared to control.	n/a	13 out of 38 patients received maintenance therapy at the time of inclusion (prednisolone, 10 mg/day, AZA or MMF, but no CYC).
**Puxeddu et al.** (53)	AAV (14)	Decreased NK cell numbers compared to controls.	Unaltered NKp46, NKp44, NKp30, NKG2D, DNAM-1, NTB-A and 2B4 compared to control.	n/a	n/a
**Tognarelli et al.** (54)	GPA (16)	Unaltered NK cell numbers and CD56^bright^ and CD56^dim^ NK cells compared to controls.	Increased CD69 and CD107a expression compared to control. Unaltered NKp30, NKp44, NKp46, NKG2D, DNAM-1, CD94, NKG2C, NKG2A and KIRs expression.		Ten patients were under standard maintenance immunosuppressive therapy (oral glucocorticoids with or without AZA). Nine patients had received RTX.
**Merkt et al.** (55)	GPA (28)	Increased NK cell numbers in remission, correlation to duration of remission. NK cell percentages positively correlate with the suppression of disease activity. Unaltered CD56^bright^ and CD56^dim^ NK cell subsets.	Unaltered expression of CD107a, NKp30, NKp46 and NKG2D on NK cells compared to control.	Unaltered degranulation upon co-culture with target cells.	All patients under DMARD therapy, 44% of the patients received continued DMARD therapy. Five out of 28 were treated with RTX.
**Braudeau et al.** (56)	AAV (41) [MPA (15) GPA (26)]	Decreased NK cell numbers in active phase which return to normal in remission in GPA and MPA.	n/a	n/a	AAV patients without any treatment in active and remission phase.
**Merkt et al.** (57)	GPA (22)	Decreased NK cell numbers in active phase and increased NK cell numbers in long-term remission. CD56^dim^ NK cells but not CD56^bright^ NK cells are decreased in active phase compared to control.	Higher expression of CD69 on CD56^dim^ CD16^+^ cells and lower expression of CD16^bright^ on CD56^dim^ CD16^+^ in active phase compared to control. Unaltered expression of NKp30, NKp44, NKp46, NKG2D, DNAM1, 2B4, CRACC and 41BB compared to control. Higher expression of CCR5, CD54 (ICAM-1) and NKG2C on CD56^dim^ cells in active phase and unaltered expression of CXCR3 compared to control.	Decreased natural cytotoxicity in active phase which returned to normal levels in remission.	Cohort I, please see Merkt, 2015 (55). Cohort II was in remission and active phase and treated with prednisone, CYC, RTX, AZA, MTX, LEF, MPA, with a combination of immune-suppressive treatment or untreated. Only AZA treatment was associated to decrease the frequency of CD16^bright^ CD56^dim^ and increase in CD69^+^ CD56^dim^ cells compared to HC.
**Fazekas et al.**(58)	AAV (38)	Unaltered NK cell numbers in active phase and remission.	n/a	n/a	Patients with and without immunosuppressive treatment, without and prior CYC treatment
**Scrivo et al.** (59)	AAV (33)	Unaltered NK cell numbers and CD56^bright^ and CD56^dim^ NK cells. Lower proportion of CD56^dim^ in ANCA-negative patients compared to controls.	Decreased TLR2 expression on NK cells in certain conditions (ANCA negative patients, on CD56^bright^ cells in ANCA positive patients and on CD56^dim^ CD57^bright^ cells). Unaltered TLR9 expression.	Unaltered IFNγ production and degranulation. Lower IL-22 levels in ANCA-positive patients compared to control.	Patients with and without immunosuppressive treatment.
**Merkt et al.** (23)	GPA (40), MPA (16), EGPA (39)	NK cell numbers are decreased in active phase in GPA, MPA and EGPA and return to normal levels in MPA patients.	n/a	n/a	Effect of AZA (n=110), MMF (n=37), MTX (n=62), RTX (n=27), and CYC (n=21) and rest (n=19) was analyzed and only AZA treatment was associated with significantly lower NK cells.

ANCA, Anti-neutrophil cytoplasmic antibody; AAV, ANCA associated vasculitis; AZA, azathioprine; CYC, cyclophosphamide; DMARD, disease-modifying anti-rheumatic drug; GPA, granulomatosis with polyangiitis; IL, interleukin; LEF, leflunomide; MMF, mycophenolate mofetil; MPA, microscopic polyangiitis; MTX, methotrexate; n/a, not analyzed; NK, natural killer; no, number; RTX, Rituximab; TLR, toll-like receptor.

Phenotype analyses of NK cells have shown more activated NK cells in AAV compared to controls, as indicated by a higher expression of the early activation marker CD69 (54, 55). The frequency of NK cells expressing the activating killer immunoglobulin-like receptor (KIR) KIR2DS3 was decreased in AAV patients compared to controls (51), but other activating and inhibitory NK cell receptors were unaltered. This included activating NKp46, NKp44, NKp30, NKG2D and DNAM-1 receptors and their co- receptors NTB-A and 2B4 (53–55), as well as activating KIRs, except KIR2D3, and inhibitory CD94/NKG2A and CD161 receptors (50, 54). The recruitment of distinct sets of NK cells to sites of infection, malignant transformation or autoimmune inflammation is guided by stimulation of cytokine and chemokine receptors expressed on the NK cells (61, 62). The analysis of chemokine receptors of NK cells, showed enhanced CCR5 expression in active phase of GPA but unaltered expression of CXCR3 and CXCR4 (50, 57). A higher expression of adhesion molecule ICAM-1 and NKG2C on CD56^dim^ cells was also observed in AAV (57). Besides KIRs, NK cells also carry TLRs to recognize or respond to pathogen-associated molecular patterns such as bacterial or viral structures leading to cytotoxicity and cytokine production. An increased expression of TLRs 2, 4 and 9 on NK cells in AAV was observed, which could be explained by a pro-inflammatory microenvironment in these patients (52). Contradictory to this publication is the expression analysis of TLR2 on different subtypes of NK cells which was lower in AAV, possibly indicating a reduced capacity to get activated, while TLR9 expression on NK cells was unaltered (59). A decreased natural cytotoxicity was furthermore observed in peripheral NK cells from GPA patients in active phase compared to control and killing correlated to the percentage of NK cells (57). However, the production of IFNγ in response to viral infection and systemic inflammation and the degranulation activity of NK cells were unaltered in AAV. This indicates that GPA NK cells are at least partially functional. There is furthermore support that GPA patient NK cells participate in cytotoxicity of cells bound Rituximab (RTX) *via* ADCC. Certain polymorphisms of FcγRIIa and FcγRIIIa are also associated with the risk for disease relapse in GPA (63), and other with the treatment response to RTX and cyclophosphamide (64).

## NK Cells in AAV Tissue

Most studies on NK cells in AAV have focused on findings in peripheral blood, however the local immune response can differ from what is observed in the circulation, making the analysis of NK cells in tissue inflammation important. A complicating factor is the lack of specific markers to identify NK cells and hence the need for more complex immunofluorescence co-staining when assessing tissue samples. There are a few studies where NK cells were detected by their expression of CD56, a marker for NK cells, but also for a subset of T cells (**Table 2**). Such basic studies detected no or rare presence of CD56^+^ cells in lung and kidney specimens of patients with GPA and pauci-immune necrotizing glomerulonephritis (55, 59, 66, 69). Using CD16 as NK cell defining marker, CD16^+^ cells were also identified in nasal tissue samples from few MPA patients with vasculitic neuropathies, however, CD16 is also expressed on neutrophils, monocytes and macrophages making the findings difficult to interpret (65).

**Table 2:** NK cells in tissue from AAV patients.

Reference	Patient group (no. of patients)	Tissue	NK cell numbers	NK cell characteristics
**Engelhardt et al.** (65)	MPA (15)	Neural	NK cells classified as CD16^+^ cells present in two out of 13 patient samples.	n/a
**Coulomb-l´Hermine et al.** (66)	GPA (6)	Lung	CD56^+^ cells rarely present in lung tissue.	High level of NK cell recruiting RANTES (CCL5).
**Capraru et al.** (67)	GPA (20)	Nasal	n/a	NK cell ligands were investigated. High level of NK cell activating IL-15. NKG2D^+^ cells MIC^+^ cells present in inflamed tissue.
**De Menthon et al.** (68)	GPA, active glomerulonephritis and remission (90)	Kidney	n/a	NK cell ligands were investigated. Strong expression of MICA/MICB and IL-15 in macrophages, interstitial infiltrates, tubular epithelial cells and glomerular mesangial cells in kidney. MICA/MICB still high in partial remission, while IL-15 expression was reduced.
**Merkt et al.** (55)	GPA (10)	Lung (12), kidney (1)	No CD56^+^ cells identified in lung or kidney tissue.	n/a
**Zhao et al.** (69)	Pauci-immune necrotizing glomerulonephritis, AAV (17)	Kidney with focal necrotizing glomerulonephritis	CD56^+^ cells rarely present in glomeruli.	CD56^+^ cells do not localize in areas with fibrinoid necrosis.
**Scrivo et al.** (59)	AAV (7)	Kidney	Rare CD56^+^ cells were found in one out of seven renal tissue samples.	n/a

AAV, Anti-neutrophil cytoplasmic antibody associated vasculitis; GPA, granulomatosis with polyangiitis; IL, interleukin; MEC, Microvascular endothelial cells; MPA, microscopic polyangiitis; n/a, not analyzed; NK, natural killer; no, number.

A possible direct role for NK cells in local cytotoxicity within the target organ was further suggested by the detection of NK cell activating ligands MICA/B and the NK cell activating cytokine IL-15 in active GPA nasal biopsies (67, 68). The low numbers of NK cells and/or their absence in AAV tissue could lead to the speculation that NK cells might not contribute to local inflammation or to granuloma formation (55, 59). However, in a GPA-like syndrome characterized by chronic granulomatous lesions in the upper respiratory tract and skin vasculitis, a strong infiltration of CD3^-^CD56^+^ NK cells into skin was observed (70). This raises the question whether NK cells could be recruited differently to different organs. In line with this possibility, microvascular endothelial cells (MECs) were used to study their response to inflammatory stimuli and transmigration of immune cells into the inflamed tissue. MECs from lung and renal specimens were stimulated with PR3 which lead to a stronger degranulation of NK cells as compared to dermal MEC´s (54). Further, renal MECs seem to be more susceptible to the proinflammatory stimuli IFNγ and TNFα leading to ICAM1, VCAM1 and CCL2 upregulation, which could result in stronger renal recruitment of NK cells. GPA-derived NK cells were also able to directly kill renal MEC´s, indicating a potential role of NK cells in GPA pathogenesis (54). No studies have so far been performed on tissue infiltrating NK cells in EGPA. Further in-depth analysis of NK cells in AAV affected tissue using for example state of the art single cell RNA sequencing or laser capture microdissection of NK cells from tissue followed by RNA or proteomic profiling would be of interest. Single cell analysis of kidney tissue of ANCA-associated glomerulonephritis exists, however, NK cells were not addressed here (71). In summary, studies on NK cells at the site of inflammation are so far few and there is a need to analyze NK cells in tissue of AAV patients to advance the field.

## The Impact of AAV Therapies on NK Cells

AAV patients are treated with glucocorticoids in combination with other immunosuppressing therapies at active flares, then followed by long-term remission maintenance therapy. To induce remission, cyclophosphamide (CYC) or RTX, an anti-CD20 chimeric antibody are commonly used (72–75). Despite intense immunosuppressive treatment, AAV patients have a high relapse rate. Studies on how NK cells are affected by immunosuppressive treatment are scarce and there is no data if they can be used to predict relapses. Recently, low peripheral NK cell numbers and percentages in GPA and EGPA, but not MPA patients, were associated with azathioprine (AZA) treatment in a dose-dependent manner, but with no other investigated immunosuppressive treatments, including mycophenolate mofetil, methotrexate, RTX and CYC (23). The lack of effect of AZA treatment on the NK cells in the MPA group could be explained by the fact that MPA patients included in this study were older than the other AAV groups and age positively correlate with NK cell number. Recently the effect of AZA treatment was analyzed in an SLE cohort and showed that these patients had an increased infection rate which was associated with AZA (76). Also, in patients with inflammatory bowel disease (IBD), AZA treatment was associated with lower NK cell counts in blood and an increased risk of herpes simplex infections, but the possible role of NK cells in that remains to be determined. In a previous study, CYC was also not shown to affect the frequency of circulating NK cells in AAV patients compared to non-CYC treated patients (23, 58). Similarly, RTX treatment or other disease-modifying anti-rheumatic drugs (DMARDs) did not alter the frequencies of NK cells in GPA patients as evaluated during different stages of the disease (55, 77). However, an analysis of the activation maker CD69 on PBMC NK cells from GPA patients prior and directly after RTX infusion showed increased activation. Furthermore, when healthy donor PBMCs were incubated overnight with RTX *in vitro*, phenotypical and functional changes of NK cells were observed (78). NK cells that were exposed to RTX showed a higher CD107a expression indicating degranulation and an enhanced activation as measured by CD69 expression on CD56^dim^ cells (79). In addition, RTX induced a down regulation of Fc-γ-receptor CD16 which is a sign of NK cell activation and enhanced the expression of the costimulatory receptor CD137 on NK cells (80) and has been implicated in the pathogenesis of autoimmunity (81). Experience from using RTX as cancer treatment suggests that NK cell counts could potentially be used as a prognostic marker for the long-term effect of the treatment. The next generation antibody against CD20, a fully humanized antibody, Obinutzumab (OBZ), showed a higher ADCC and direct B cell killing (82). A study comparing OBZ to RTX showed that OBZ activates NK cells from GPA patients more efficiently and increases the depletion of non-malignant B cells in *in vitro* setting overnight (79). In conclusion, the effect of treatment on NK cells must be consideration as an important factor when assessing NK cell numbers, phenotype and function in AAV. Conversely, NK cells can potentially also participate in the effects of therapies, including cell-binding antibodies.

## Concluding Remarks and Future Perspectives

AAV is a group of complex systemic autoimmune disease involving several target organs. This makes it challenging to firmly conclude about the properties and potential role of NK cells in the disease pathogenesis. NK cells in blood of AAV patients have an altered and activated phenotype, but whether the number and frequency of NK cells are also affected is not conclusively determined. Most published studies suggest that number or percentages of circulating NK cells in AAV patients are unaltered but other studies report an increase or decrease, especially in the active phase of the disease. NK cell numbers and frequency are however difficult to interpret, since there is a vast heterogeneity also in healthy controls. Furthermore, AAV patients’ treatments could potentially impact NK cells in AAV, and evidence for this is particularly strong for AZA (23). A potential role for NK cells in the effect of cell-binding therapeutic antibodies, *via* ADCC can also be envisioned. NK cells are also able to infiltrate into the target organ, but in low numbers and the biological significance of these cells or their role in the pathogenesis of AAV remains to be explored, by for example single cell analysis. Novel techniques for spatial analysis of protein and RNA expression in tissue sections could further advance the field and increase the knowledge about the immunological landscape in AAV organs, including NK cells.

## Author Contributions

SF, AS, and HB wrote the manuscript. IG gave clinical input. All authors participated in discussing and finalizing the manuscript. All authors contributed to the article and approved the submitted version.

## Funding

This work was supported by Åke Wiberg Foundation [M19-0665]; Cancerfonden [19 0408 Fk]; Hudfonden [3147]; Magnus Bergsvall Foundation [2019-03538]; Clas Groschinskys Foundation [M19390]; Stockholm county council/Region Stockholm [20190859] and the Swedish Society for Medical Research [S17-0104] to HB, and Karolinska Institutet Foundations to HB [2018-02203, 2018-02839] and SF [2019-01970].

## Conflict of Interest

The authors declare that the research was conducted in the absence of any commercial or financial relationships that could be construed as a potential conflict of interest.

## Publisher’s Note

All claims expressed in this article are solely those of the authors and do not necessarily represent those of their affiliated organizations, or those of the publisher, the editors and the reviewers. Any product that may be evaluated in this article, or claim that may be made by its manufacturer, is not guaranteed or endorsed by the publisher.
